# dbDEPC 3.0: the database of differentially expressed proteins in human cancer with multi-level annotation and drug indication

**DOI:** 10.1093/database/bay015

**Published:** 2018-02-22

**Authors:** Qingmin Yang, Yuqi Zhang, Hui Cui, Lanming Chen, Yong Zhao, Yong Lin, Menghuan Zhang, Lu Xie

**Affiliations:** 1 College of Food Science and Technology, Shanghai Ocean University, Shanghai 201306, China; 2 Shanghai Center for Bioinformation Technology, Shanghai Academy of Science and Technology, Shanghai 201203, China; 3 School of Medical Instrument and Food Engineering, University of Shanghai for Science and Technology; 4 School of Life Science and Technology, ShanghaiTech University, Shanghai 201210, China; 5 Institute for Nutritional Sciences, Shanghai Institutes for Biological Sciences, University of Chinese Academy of Sciences, Shanghai 200031, China; 6 Laboratory of Quality and Safety Risk Assessment for Aquatic Products on Storage and Preservation (Shanghai), Ministry of Agriculture, Shanghai 201306, China; 7 Shanghai Engineering Research Center of Aquatic-Product Processing and Preservation, Shanghai 201306, China

## Abstract

Proteins are major effectors of biological functions, and differentially expressed proteins (DEPs) are widely reported as biomarkers in pathological mechanism, prognosis prediction as well as treatment targeting in cancer research. High-throughput technology of mass spectrometry (MS) has identified large amounts of DEPs in human cancers. Through mining published researches with detailed experiment information, dbDEPC was the first database aimed to provide a systematic resource for the storage and query of the DEPs generated by MS in cancer research. It was updated to dbDEPC 2.0 in 2012. Here, we provide another updated version of dbDEPC, with improvement of database contents and enhanced web interface. The current version of dbDEPC 3.0 contains 11 669 unique DEPs in 26 different cancer types. Multi-level annotations of DEPs have been firstly introduced this time, including cancer-related peptide amino acid variations, post-translational modifications and drug information. Moreover, these multi-level annotations can be displayed in the biological networks, which can benefit integrative analysis. Finally, an online enrichment analysis tool has been developed, to support a KEGG enrichment analysis and to browse the relationship among interested protein list and known DEPs in KEGG pathways. In summary, dbDEPC 3.0 provides a comprehensive resource for accessing integrated and highly annotated DEPs in human cancer.

**Database URL**: https://www.scbit.org/dbdepc3/index.php

## Introduction

Proteins are major effectors of biological functions, and differentially expressed proteins (DEPs) are widely reported as biomarkers in pathological mechanism, prognosis prediction as well as treatment targeting in cancer research. For example, DEP APOBEC was identified as an oral cancer prognostic biomarker, based on the quantitative proteomics experiment on Taiwanese Carriers of an APOBEC deletion polymorphism (1). Generally, the analysis of DEPs is an indispensable approach in cancer research.

**Figure 1.**
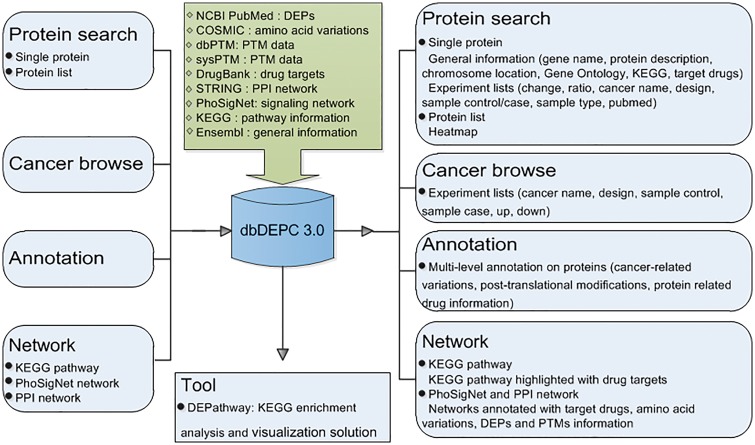
Overview of database construction. Query methods and corresponding output information are demonstrated.

Mass spectrometry (MS) is the core technology in the field of high throughput proteomics. It identifies large amounts of DEPs data every year, scattered in a wealth of peer reviewed MS/MS literatures. Thus, it is of great necessity to catalogue them in a favorable way, helping to efficiently and conveniently extract meaningful biological insights from the sporadic DEPs data. With this consideration, we published the first proteomics database in human cancer (dbDEPC) in 2009 (2), and an updated version was provided in the year of 2012 (dbDEPC 2.0) (3). Since then, a series of cancer proteome databases have been established. For instance, DBGC (4) collected DEPs focused on gastric cancer-related transcriptomic and proteomic data. PED (5) and PCD (6) are both the databases of pancreatic cancer containing multi-dimensional data types pertaining to quantitative alterations. Colorectal Cancer Atlas (7) is a web-based resource which catalogues genomic and proteomic data from CRC tissues and cell lines. BCCTBbp (8) stores omics data involved in breast cancer. The Cancer Proteomics database (9) is dedicated to proteome study on prostate cancer. Most of the databases above provide comprehensive omics data targeted at one kind of cancer, and only the Cancer Proteomics database is a proteome oriented database for prostate cancer.dbDEPC is the only repository currently available for storing MS-based DEPs in human cancers, characteristic with proteome data in various types of cancer. In recent years, with the rapid development of MS technology, a huge amount of proteomic data have been generated, which motivates an update on dbDEPC. In this version of dbDEPC 3.0, data content has expanded to 11 669 unique DEPs covering 26 different cancer types, extracted from 779 MS experiments and all the data types have been increased more than twice compared to the previous version. Also, the web interface has been greatly refined and enhanced. On the one hand, to characterize the proteomic alterations in different aspects will promote a better understanding of molecular drivers of cancers, so we introduced multi-level annotation on DEPs, including peptide amino acid variation and protein post-translational modifications (PTMs) information. On the other hand, to allow for the advantage of network approaches in the identification of drug target (10), we integrated targeting drug information with different biological networks, expecting to shed light on network-based drug annotation and drug discovery (Figure 1). Finally, an online tool has been developed in dbDEPC 3.0 to help access molecular interaction and reaction networks for DEPs in KEGG pathway.

## Materials and methods

### Data collection and processing

The dbDEPC 3.0 mainly consists of two datasets (Table 1). The main dataset is the DEPs, which are derived from peer reviewed MS/MS literature. The other dataset is the annotation information of DEPs, which is derived from public data repositories. Detailed data collection procedure is as follows:

**Table 1.** Data sources of dbDEPC 3.0

Sources	Data type	URL
NCBI PubMed	DEPs	https://www.ncbi.nlm.nih.gov
COSMIC	Mutations	http://cancer.sanger.ac.uk/cosmic/ download
dbPTM	PTMs	http://dbptm.mbc.nctu.edu.tw/ download.php
sysPTM	PTMs	http://lifecenter.sgst.cn/SysPTM/
DrugBank	Drug targets	https://www.drugbank.ca/
Ensembl	General information	http://asia.ensembl.org/index.html
KEGG	Pathway information	http://www.kegg.jp/kegg/pathway. html
PhoSigNet	Signaling network	http://lifecenter.sgst.cn/PhoSigNet/
STRING	PPI network	https://string-db.org/

#### Data of DEPs

- A text mining was conducted on PubMed abstracts using proteomics keywords (quantitative proteomics, proteome), MS-related words (MS), keywords describing expression changes (upregulated, down-regulated, differentially expressed and fold change), restriction of species (human cancer). The current version of dbDEPC indexed papers published between April 2011 and June 2016.
- To control the data quality, manual check of the retrieved papers was conducted as described in our previous versions (2, 3). Simply, during data collection, proteins with expression changes are defined as DEPs among different samples, such as diseased vs. healthy tissue or treated vs. non-treated tissue. Then, DEPs must meet the differential expression filtering criteria, which is a strict score cutoff or *P*-value of a statistical test provided in the original experiment.
- For the purpose of unifying the identifiers and names of DEPs from papers, ID Mapping Service at UniProt (11) was used to match different protein ID numbers to UniProtKB accession numbers.
- The original cancer names from papers were unified according to the standardized names provided by NCBI MeSH (https://www.ncbi.nlm.nih.gov/mesh).
- Experimental descriptions were refined as the PRIDE database (12), in order to display an intuitive browsing of the experimental information.

#### Data of annotation

To provide additional annotation for each DEP, new external resources were integrated including general information, cancer-related variations, PTMs and protein related drug information.

General information was downloaded from the Ensembl database through BioMart (13), including gene name, protein description, chromosome location, Gene Ontology (GO) annotations, Pfam domains, amino acid change conservation and different protein/gene identifiers. Pathway information was collected from KEGG (14). Human protein − protein interaction network was downloaded from STRING (15) and human phosphorylation mediated signaling transduction network was extracted from in-house constructed PhoSigNet (16).

Both protein sequence variations and expression changes are important molecular phenotypes in human cancer, and they have often been used as cancer biomarkers or drug targets. The somatic mutation information was collected from COSMIC database (17). Four types of gene point mutation were chosen, including missense substitution, non-sense substitution, in-frame deletion and in-frame insertion. Furthermore, the cancer names were uniformed to the standardized names provided by NCBI MeSH, based on the primary histology of the sample.

As we know, one protein can have multiple PTM sites; such rich PTM sites may constitute the modification switches of a signaling network. Therefore, it is meaningful to integrate protein with variations, protein with expression changes and protein with PTM into biological pathways or protein-protein interaction networks. Hence, we acquired experimentally verified PTM data from dbPTM and SysPTM, which are comprehensive and informative resources for protein PTMs (18, 19).The modification annotation types in dbDEPC 3.0 included: phosphorylation, acetylation, methylation, ubiquitylation and glycosylation (N, O, C-linked).

In order to find out whether these abnormal proteins in cancers are already targets for known drugs, drug target identifiers were extracted from DrugBank, which is a database containing extensive biochemical and pharmacological information about drugs and their targets (20).

### Database construction

dbDEPC 3.0 consists of a relational database and a dynamic web interface. The framework of the database and web server is shown in Figure 1. The database was implemented using MySQL5 (http://www.mysql.com/). The web interface was implemented with PHP technology. The KEGG pathway enrichment analysis was developed with Python. The network tool was developed with Cytoscape.js (http://js.cytoscape.org/).

## Results

### Improvement of database contents

**Figure 2.**
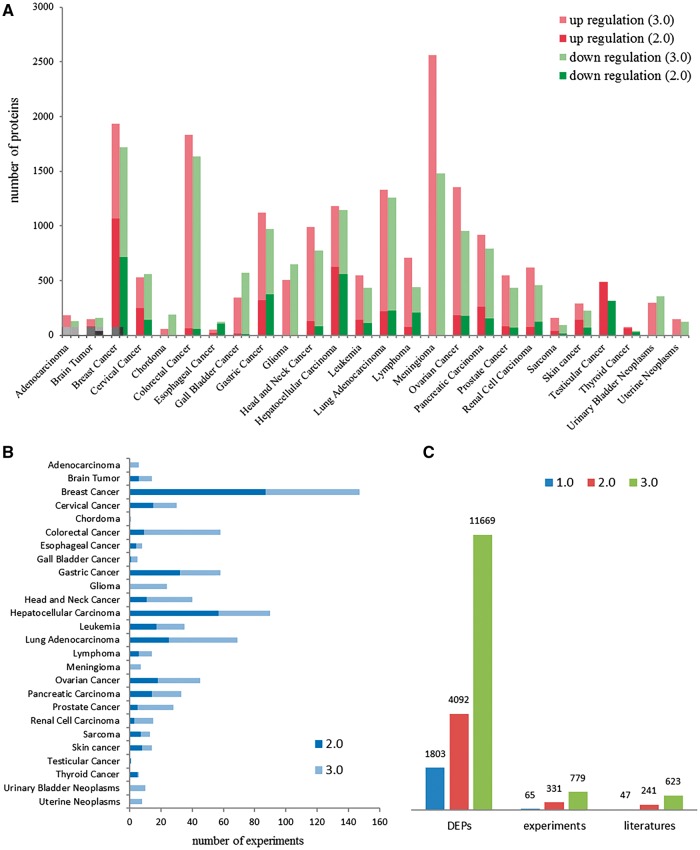
Data contents in dbDEPC 3.0. (**A**) The distribution of DEPs in each cancer in version 2.0 and 3.0. (**B**) Number of experiments in each cancer. (**C**) Number of DEPs, MS experiments and related literatures in three version of dbDEPC.

dbDEPC was designed for the storage and query of the DEPs in human cancers. In this current version, dbDEPC 3.0 contains 11669 unique DEPs in 26 different cancer types and 28 subtypes. Adenocarcinoma, glioma, meningioma, urinary bladder neoplasms, uterine neoplasms and chordoma are the six new cancer types included in this version (Supplementary Table S1). dbDEPC 3.0 now has documented 779 MS experiments from 623 peer-reviewed papers. The numbers of DEPs in meningioma, colorectal cancer and breast cancer are augmented dramatically during the past 5 years (Figure 2A). Lots of MS experiments are related with breast cancer, hepatocellular carcinoma and lung adenocarcinoma (Figure 2B). Overall, data content of the unique DEPs, MS experiments and related literatures have sharp increments by 2.9, 2.4 and 2.6 times, respectively, compared to version 2.0 (Figure 2C). Additionally, multi-level annotation information, including 2 130 668 cancer-related protein variations (crVARs), 110 343 PTMs and 4908 items of protein related drug information were also integrated into dbDEPC 3.0. Supplementary Figure S1 illustrates DEPs in each cancer and the number of mutations and PTMs on DEPs.

### New features

In dbDEPC 2.0, different query ways were provided, for example, protein query, cancer type query and MS experiments browse. In version 3.0 here, the web interface have been significantly refined and improved, to facilitate the better use of the data. Multi-level annotations of DEPs have been firstly introduced, including cancer-related variations, PTMs and drug information. Moreover, these multi-level annotations are displayed in different biological networks, including KEGG, STRING and PhoSigNet, which can benefit integrative analysis. Finally, an online enrichment analysis tool has been developed, to support a KEGG enrichment analysis and to browse the relations between interested protein list and known DEPs in KEGG pathways.

#### Enhanced web interface

**Figure 3.**
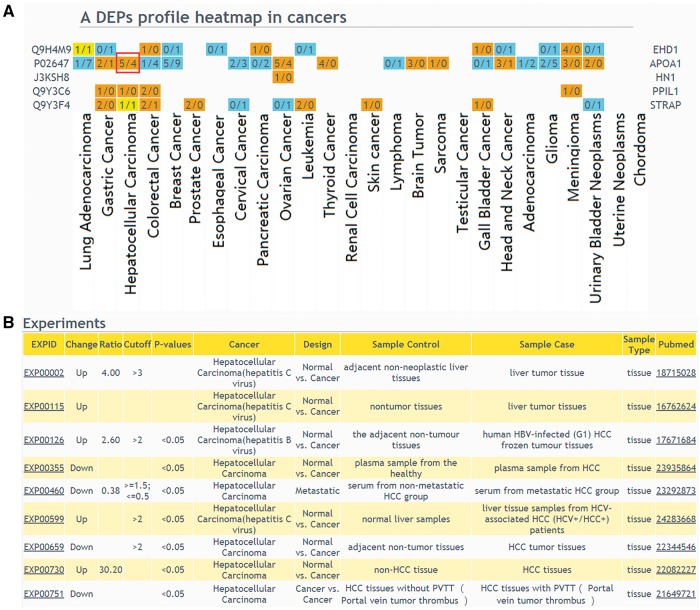
The result of ‘protein list’ query. (**A**) Users should select a design type of interested experiments first, input protein IDs or names and then select cancers to draw a DEPs profile heatmap in cancers. If a queried protein is a DEP, the table cell will be displayed in color. Orange means that the experiments number identified this protein as up-regulated is more than the experiments number identified protein as down-regulated; blue means that the experiments number identified this protein as up-regulated is less than the experiments number identified protein as down-regulated; yellow means that the experiments number identified the protein as up-regulated is equal to the experiments number identified protein as down-regulated. (**B**) If the number of experiments in a specific cancer type is clicked, the detailed information of these DEPs can be displayed.

The web interface of browsing and querying DEPs has been greatly improved. Protein search page now allows users to extract summarized information of a protein or protein lists. Using a single protein query, general information of the interested protein will be retrieved, including protein description, chromosome location, GO, KEGG pathway and drug annotation. Importantly, DEP related experiments will be described. In protein list query, an user should select a design type of your interested experiment first, input protein IDs or names and then select cancers to draw a DEPs profile heatmap in cancers (Figure 3A), which shows protein up expression (orange) or down expression (blue) decided by whether the upregulation or downregulation results are the major (numbers in brackets). If the number of experiments in a specific cancer type is clicked, the detailed information of these DEPs can be displayed (Figure 3B).

The ‘Cancers’ query allows users browse experiments and DEPs in dbDEPC. To accommodate elaborate and precise queries, the ‘Cancers’ page provides user with the filter tools, namely cancer type, sample type and experimental design. After filtering the experimental results, users can select interested experiments and click the button of ‘View’ to have an overview of the DEPs in the selected experiments. These DEPs can be downloaded for further analysis. Thus, users can have more control over the selection of specimen/sample information and DEPs can be batch extracted.

#### Multi-level annotation

**Figure 4.**
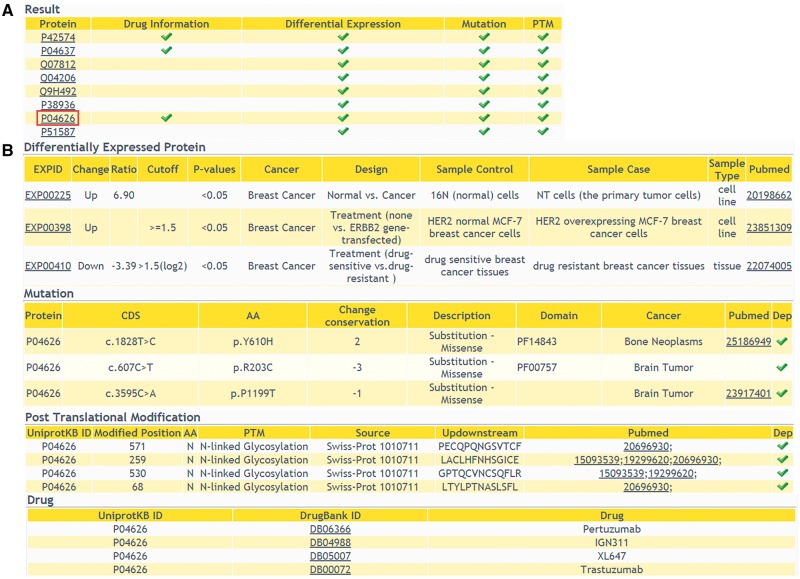
The returning page of ‘Annotations’ query way. (**A**) The status of multiple-level annotation is showed in a table. (**B**) The detailed information can be displayed with a further click.

In addition to providing comprehensive information of DEPs, we also allow users to browse multi-level annotation of interested proteins, including cancer-related variations, PTMs and protein related drug information. In ‘Annotations’ query way, a list of candidate proteins should be submitted. The result shows that whether the queried proteins have multi-level annotations (Figure 4A). Detailed information of the proteins can be retrieved with a further click (Figure 4B). This comprehensive annotation helps users to browse more molecular biological data, especially cancer-related data, in one web interface. It facilitates the integration of data at different levels.

#### Drug indication

**Figure 5.**
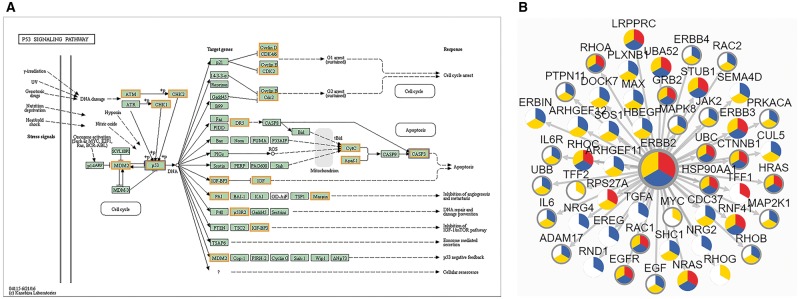
The result of ‘Networks’ query way. (**A**) The result of ‘KEGG Pathway’ query. Candidate proteins should be entered first and KEGG pathway ID should be selected. A colored KEGG pathway will be drawn with drug targets highlighted in the result. Orange means drug targets in our database, blue means the queried proteins. (**B**) The result of ‘PPI’ query. The page demonstrates association network of ERBB2 in Breast cancer with 900 score threshold of network edge and multi-level annotation of network node. Nodes are divided into three colored sectors. Red means the protein is differentially expressed. Blue means the protein is with variations. Yellow means the protein has PTMs. The gray edge means that this protein is a drug target.

Another new feature we would like to point out is the potential drug target discovery based on network. As is known, proteins can be involved in multiple life activities and communicate with each other in biological system. In this version, biological pathway, protein–protein interaction (PPI) and phosphorylation-mediated signaling transduction networks (PhoSigNet) are provided as representative molecular networks. In ‘KEGG Pathway’ query, candidate proteins should be entered first and KEGG pathway ID should be selected. A colored KEGG pathway will be drawn with drug targets highlighted in the result. Figure 5A illustrates drug information involved in P53 signaling pathway. In ‘PPI’ query, users should input an interested protein first, set an score (0–1000) threshold of network edge, select one or more cancer types and then select annotation types of network node. The query result shows a network of the first-layer-associated proteins of the interested protein node in dbDEPC. The multi-level information will be highlighted, including target drugs information, differentially expression information, protein variations and PTM information (Figure 5B). Moreover, the detailed information can be shown with clicking on the network node. The ‘PhoSigNet’ query result is similar with ‘PPI’ query result. In short, annotation information can be integrated in the pathways or networks, which can help accelerate the research of multi-level data and drug targets integration.

#### Enrichment analysis tool of DEPathway

Pathway analysis could demonstrate on how the DEPs coordinate in cell signaling. DEPathway enrichment algorithm in dbDEPC 3.0 is designed based on hypergeometric analysis. The function of hypergeometric distribution is:

PX=k=MkN-Mn-kNn

Here, *N* is the population of the total proteins in KEGG pathways. *M* is the number of proteins involved in specific pathway. The *n* represents the number of queried proteins. The *k* is the number of proteins in n involved in a specific pathway. ab is a binomial coefficient.

**Figure 6.**
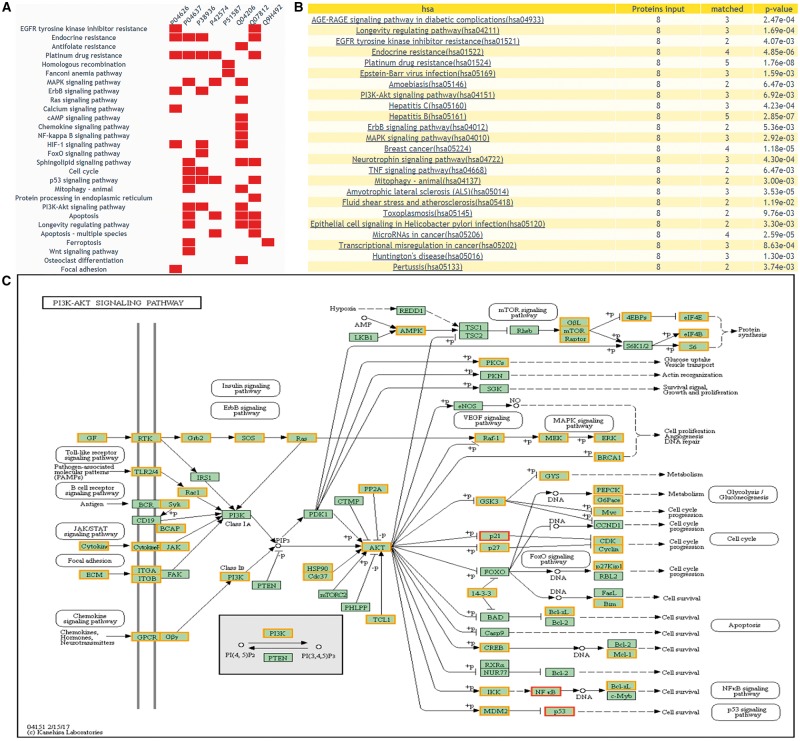
The returning page of enrichment analysis. (**A**) A heatmap is drawn showing the distribution of queried proteins in pathways. (**B**) The table lists those enriched pathways with a threshold of *P*-value <= 0.05. (**C**) DEPs on KEGG pathway maps. Blue means query proteins. Orange means the DEPs in our database and red means both DEPs and query proteins.

DEPathway provides a visualization solution to characterize the DEPs in the pathway. The distribution of the queried proteins in KEGG pathways will be shown, with differential expression information highlighted. This function can help users clearly see which pathway is enriched with their candidate proteins and whether these candidate proteins have been reported as DEPs of human cancers in previous proteomics research. Figure 6 illustrates the result of ‘DEPathway’ enrichment analysis.

## Discussion

dbDEPC 3.0 is committed to be a practical database in the study of cancer proteomics. It allows researchers to exploit huge volumes of DEPs data. And we expect it can provide guidance on experimental design and data analysis.

**Figure 7.**
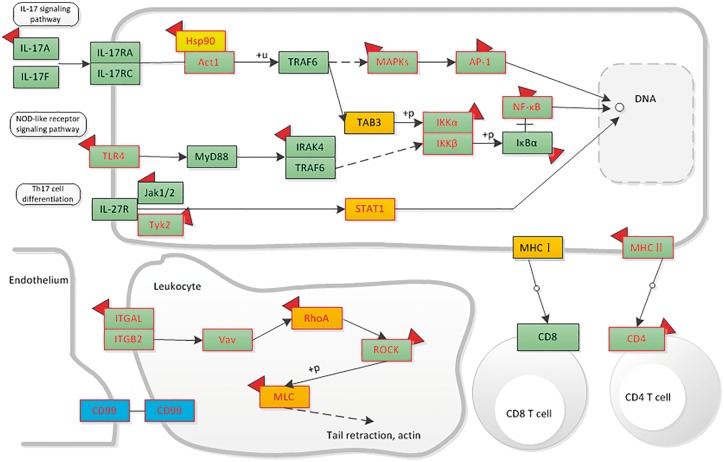
The distribution of liver-associated proteins in immune system related signaling networks. Orange means the up-regulated protein, blue means the down-regulated protein. Protein with mutation information is colored by red frame. The drug targets are attached with a red symbol.

Here, we give an example of how dbDEPC could help in cancer research. Given that a researcher would like to study DEPs in hepatocellular carcinoma, he/she could take the steps as below in our database. First, he/she should turn to ‘Cancers’ navigation bar to search for all the relevant DEPs. Through selecting the cancer type of ‘Hepatocellular Carcinoma’, clicking the experiment design of ‘Normal vs. Cancer’ and filter the sample type by ‘tissue’, 51 eligible experiment lists would be displayed. To retrieve all the DEPs, he/she should select all the experiment IDs. With a further click of ‘View’ button, a total of 2520 protein information lists are accessible for download, including 729 up-regulated proteins, 794 down-regulated proteins (380 proteins are both up and down-regulated). Then, we use ‘DEPathway’ tool with all the acquired DEPs for KEGG enrichment analysis. We find that the up-regulated proteins are enriched in 15 pathways, mainly related to immune system, transcription and carbohydrate metabolism; while the down-regulated proteins are enriched in 57 pathways, most of these pathways are metabolic-related pathways, such as lipid metabolism, amino acid metabolism. Particularly, among these pathways, we could integrate signaling pathways involved in the immune system and highlight the mutation, DEPs and drug information to help identify or predict biomarkers in hepatocellular carcinoma. Through the up- and downstream pathway, the relationship among molecular markers and drug indication could also be discovered (Figure 7).

More general analyses could also be performed. It could be seen, in some cases, specific proteins show different behavior in different cancers. To draw some general conclusion, we performed consistency analysis based on DEPs of the design ‘Normal vs. Cancer’. We found that, with overall analysis, some proteins may show a preferred expression tendency. For instance, the protein of ‘NPM1’ shows upregulation in most of the cancers, it was only identified as down-regulated in Head and Neck Cancer. With the ratio of 92.8% upregulation and 7.2% downregulation, we can infer that ‘NPM1’ may act as an upregulated protein in cancer progression. A contrary example is, the protein ‘COL14A1’ shows downregulation percentage of 88.9% and upregulation percentage of 11.1% in all cancer types. Moreover, the enrichment analysis of the top 20 proteins with high percentage of upregulation indicates that up regulated proteins are mainly associated with biological process of mRNA splicing and transcription. The enrichment analysis of the top 20 proteins with high percentage of down regulation indicates that down-regulated proteins are in connection with metabolism and oxidative phosphorylation (Supplementary Figures S2 and S3). In a word, by performing the consistency analysis, we may be able to find the preferred behavior of specific proteins and the common characteristics of DEPs in human cancers. Similar topic focused cross-cancer analysis may be performed based on data in dbDEPC 3.0.

Overall, we expect that dbDEPC 3.0 could serve as a systematic resource, with comprehensive data amount and data analysis tool to facilitate study of DEPs in human cancer. We also see the potential needs of continuous updates and improvements that have to be met in the future. We believe that the development of dbDEPC database can help accelerate the integration between proteomic and cancer studies.

## Supplementary data

Supplementary data are available at *Database* Online.

## Funding

This work was supported by National Key Research and Development Program of China [2016YFC0904101]; National Natural Science Foundation of China [31570831]; National Hi-Tech Program [2015AA020101] and Chinese Human Proteome Projects [CNHPP: 2014DFB30020, 2014DFB30030].

*Conflict of interest*. None declared.

## Supplementary Material

Supplementary Data
