# Supplementary material for: dbDEPC 3.0: the database of differentially expressed proteins in human cancer with multi-level annotation and drug indication
Source: Database (Oxford). 2018 Feb 22;2018:bay015. doi: 10.1093/database/bay015 (PMC5824774; doi:10.1093/database/bay015)
Supplement: Supplementary Data [file bay015_supp.doc]

**Table S1.** Cancer Types in dbDEPC 3.0

| Cancer Type | Subtype |
| --- | --- |
| Lung Adenocarcinoma | non-small cell lung carcinoma |
|  | small cell lung carcinoma |
| Hepatocellular Carcinoma | hepatitis B virus |
|  | hepatitis C virus |
| Breast Cancer | breast ductal carcinoma |
|  | triple negative breast cancer(TNBCs) ** |
| Pancreatic Carcinoma | pancreatic ductal adenocarcinoma |
|  | insulinoma** |
| Leukemia | chronic myeloid leukemia |
|  | chronic lymphocytic leukemia |
|  | acute myeloid leukemia |
|  | acute lymphoblastic leukemia |
| Thyroid Cancer | papillary thyroid carcinoma |
|  | follicular thyroid carcinoma |
|  | follicular thyroid adenoma |
| Skin cancer | melanoma |
|  | nonmelanoma |
| Lymphoma** | non-Hodgkin's Lymphoma |
|  | Hodgkin's Lymphoma |
|  | neuroblastoma |
| Sarcoma** | osteosarcoma |
|  | fibrosarcoma |
| Head and Neck Cancer | oral Cancer |
|  | oral premalignant lesions |
|  | laryngeal cancer** |
|  | nasopharyngeal carcinoma** |
| Glioma** | glioblastoma |
|  | oligodendroglioma |
| Gastric Cancer |  |
| Colorectal Cancer |  |
| Prostate Cancer |  |
| Esophageal Cancer |  |
| Cervical Cancer |  |
| Ovarian Cancer |  |
| Renal Cell Carcinoma |  |
| Brain Tumor |  |
| Testicular Cancer |  |
| Gall Bladder Cancer |  |
| Adenocarcinoma** |  |
| Meningioma** |  |
| Urinary Bladder Neoplasms** |  |
| Uterine Neoplasms** |  |
| Chordoma** |  |

**Marked the new human cancer types and subtypes in dbDEPC 3.0


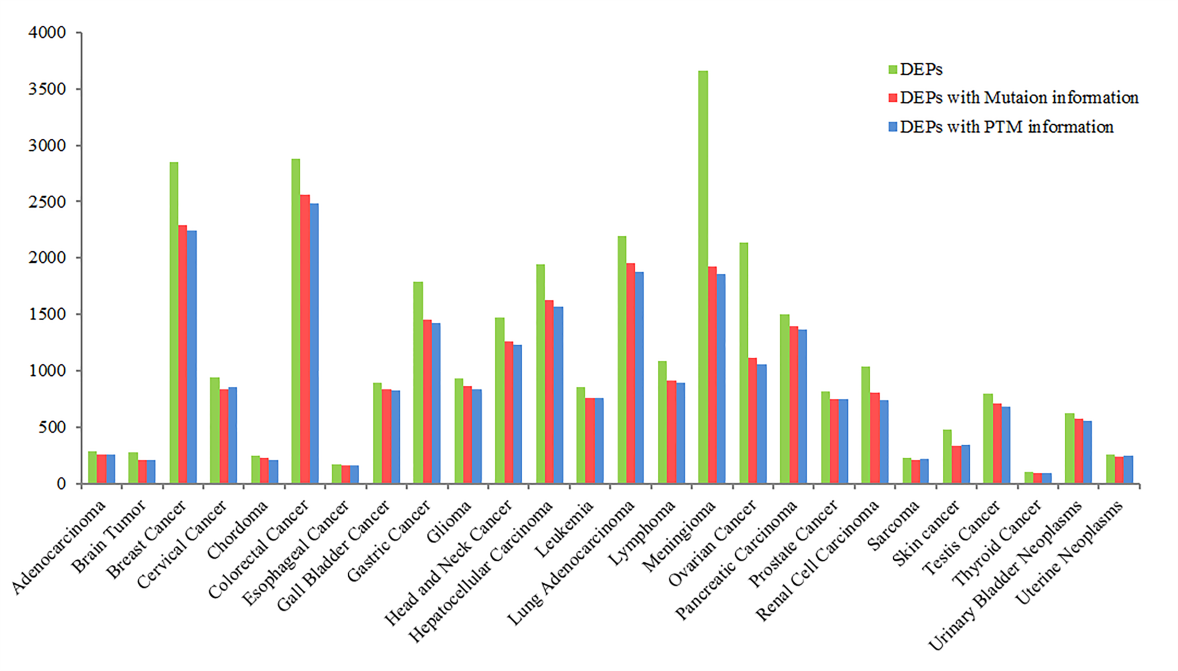


**Figure S1.** Number of DEPs in each cancer and number of the mutations and PTMs on DEPs.


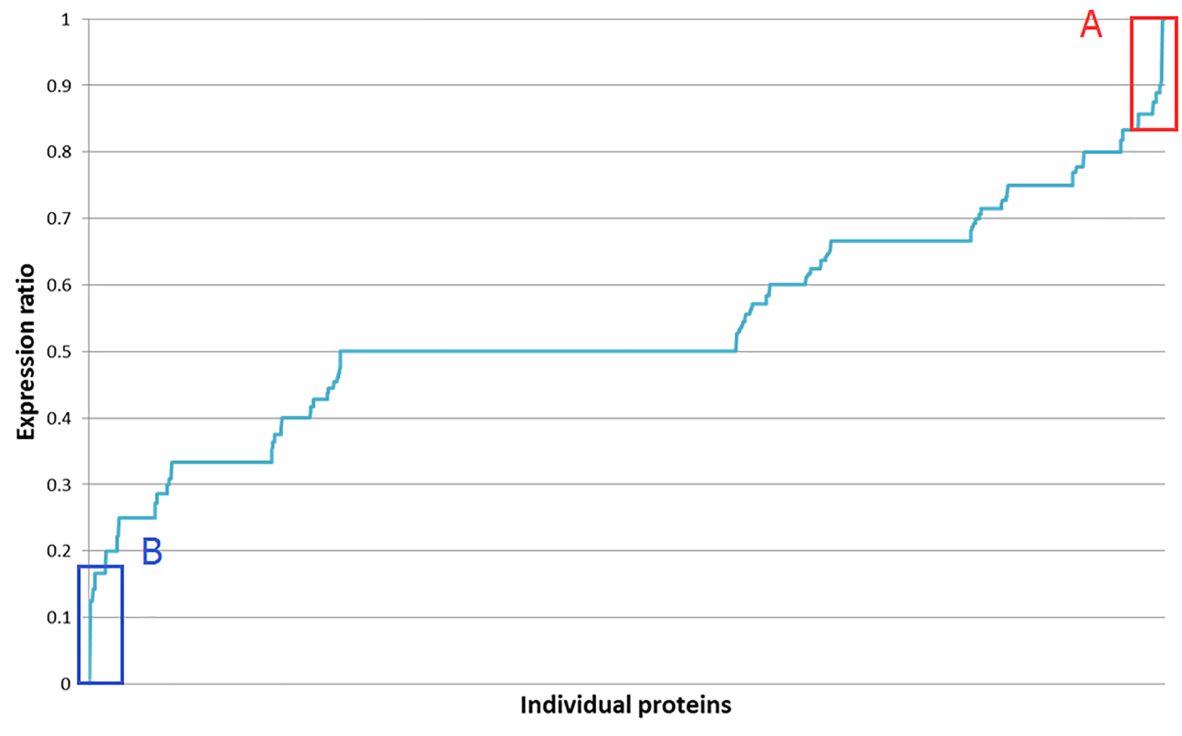


**Figure S2.** The expression preferance of individual proteins, a ‘ratio’ indicates the percentage of cancer type number with a protein showing up-regulation to total cancer type number with expression of that protein. Square A are the top twenty proteins with high percentage of upregulation and square B are the top twenty downregulated proteins in human cancers.


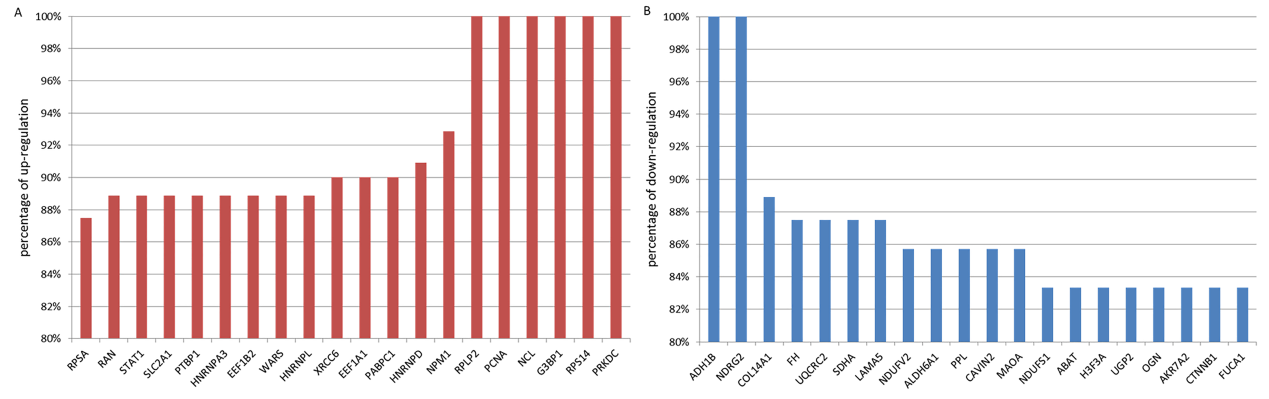


**Figure S3.** The top 20 proteins with high percentage of upregulation (A) and downregulation (B) in human cancers
